# Local Abundance Patterns of Noctuid Moths in Olive Orchards: Life-History Traits, Distribution Type and Habitat Interactions

**DOI:** 10.1673/031.011.0132

**Published:** 2011-03-18

**Authors:** Sergio Pérez-Guerrero, Alberto José Redondo, José Luis Yela

**Affiliations:** ^1^ Departamento de Ciencias y Recursos Agrícolas y Forestales Área de Entomología Agroforestal. ETSIAM.Universidad de Córdoba, Edificio Celestino Mutis. Campus Universitario de Rabanales. Crta. Madrid-Cádiz, km. 396-a, 14071, Córdoba, Spain; ^2^ Departamento de Zoología, Facultad de Ciencias, Universidad de Córdoba, Edificio Darwin. Campus Universitario de Rabanales. Crta. Madrid-Cádiz, km. 396-a, 14071, Córdoba, Spain; ^3^ Área de Zoología, Facultad de Ciencias del Medio Ambiente, Universidad de Castilla-La Mancha, Avda. Carlos III s/n. Edificio Sabatini, Despacho 0.26, E-45005 Toledo, Spain

**Keywords:** agro-ecosystem, dispersal ability, distribution, feeding specificity, phylogenetic comparative methods

## Abstract

Local species abundance is related to range size, habitat characteristics, distribution type, body size, and life-history variables. In general, habitat generalists and polyphagous species are more abundant in broad geographical areas. Underlying this, local abundance may be explained from the interactions between life-history traits, chorological pattern, and the local habitat characteristics. The relationship within taxa between life-history traits, distribution area, habitat characteristics, and local abundance of the noctuid moth (Lepidoptera: Noctuidae) assemblage in an olive orchard, one of the most important agro-ecosystems in the Mediterranean basin, was analyzed. A total of 66 species were detected over three years of year-round weekly samplings using the light-trap method. The life-history traits examined and the distribution type were found to be related to the habitat-species association, but none of the biological strategies defined from the association to the different habitats were linked with abundance. In contrast to general patterns, dispersal ability and number of generations per year explained differences in abundance. The relationships were positive, with opportunistic taxa that have high mobility and several generations being locally more abundant. In addition, when the effect of migrant species was removed, the distribution type explained abundance differences, with Mediterranean taxa (whose baricenter is closer to the studied area) being more abundant.

## Introduction

Many ecological studies have shown differences between the characteristics of abundant and rare species (Kunin and Gaston 1993; Gaston 1994; Blackburn et al. 1996; Blackburn et al. 2006; Freckleton et al. 2006; Zuckerberg et al. 2009). In general, abundant species have a broader distribution range, and more dense populations tend to be located towards the center of the distribution area (Brown 1984; Blackburn 1991; Lawton 1993; Beck et al. 2006; Freckleton et al. 2006; Zuckerberg et al. 2009). Nevertheless, conclusions and patterns found could be scale-, taxon-, and habitat-dependent (Gaston and Lawton 1990; Fangllang and Gaston 2000; Cowley et al. 2001; Blackburn et al. 2006). Life-history traits of species can also determine differences in abundance (Gaston and Lawton 1988a; Inkinen 1994; Blackburn et al. 1996; Blackburn et al. 1997; Quinn et al. 1997; Eriksson and Jakobsson 1998; Zuckerberg et al. 2009). In these studies, results differed according to the taxon and variables selected. So, Blackburn et al. (1996) found that both life span and lifetime reproduction in British birds determined abundance when phylogenetic relationships between different species of birds were considered. Studies with macrolepidoptera showed that only habitat generalism and degree of polyphagy significantly explained the variation in abundance (Quinn et al. 1997). In particular, these authors found that generalist and polyphagous species were more abundant. In contrast, for bracken herbivores, Gaston & Lawton (1988a) showed that polyphagous species were scarcer. However, this study did not take phylogeny into account.

Some researchers have evaluated the relationship of abundance and life history traits within the Noctuidae. Inkinen (1994) showed that the most abundant species were generalists and polyphagous. Rejmánek and Spitzer (1982) positively correlated variation in annual abundance, degree of polyphagy, voltinism, and dispersal ability of noctuid moths [although for variation in annual abundance and degree of polyphagy, the results have been refuted by other authors such as Nieminen (1996) and Gaston and Lawton (1988b)]. In none these works were the effects of phylogeny controlled for (Harvey and Pagel 1991).

Overall, studies examining effects of life history traits on abundance have focused on general abundance patterns in large geographical areas, including a wide variety of ecosystems. Thus, very few previous papers focusing on this pattern in a particular agro-ecosystem have been found. Lozosova et al. (2008) evaluated which biological traits, ecological characteristics, and distributional characteristics were most closely related to the regional abundance of weed species on a wide variety of arable land such as cereal, root crops, uplands, and lowlands. These authors found that the most important attributes are those that enable weeds to grow and reproduce in the cool season when there is limited competition with crop plants, and those that enable them to growth in dense vegetation stands and highly productive habitats. In addition, some works have tested the effects of agricultural intensification on abundance-life history traits relationships (Burel et al. 2001; Jennins and Pocock, 2009). Burel et al. (2001) showed that dispersal ability and body size of Diptera and Coleoptera, respectively, determine differences in abundance under different conditions of landscape context and agricultural intensification. Jennins and Pocock (2008) found that some ecological traits of insectivorous mammals and arthropods, associated with fast life histories and low mobility, were related with the sensibility to agricultural intensification. The present study evaluates life-history traits, distribution type and habitat interactions of noctuid moths (Lepidoptera: Noctuidae) in relation with local abundance patterns, in particular, in olive orchards in South Spain.

Olives are one of the major crops in the Mediterranean basin. Several studies have evaluated the biology of insect pests that damage the crop significantly (Ramos et al. 1998; Broumas et al. 2002; Shehata et al. 2003). Nevertheless, studies on non-pest, olive orchard-based resident or transient arthropod assemblages are scarce (Morris and Fields 1999; Morris et al. 1999; Ruano et al. 2004). This agro-ecosystem represents a dominant, continuous landscape where natural vegetation is almost absent except ephimeral weeds, causing drastic variations in food availability and refuges against natural enemies. Thus, these unstable environmental situations may be compensated by the occupation of olive orchards by species tending to show more opportunistic (generalistic) strategies, which theoretically would translate in higher egg number production, higher dispersal ability, higher degree of polyphagy, and higher number of generations per year (see Rejmánek and Spitzer 1982; Inkinen 1994; Quinn et al. 1997). Our hypothesis, therefore, is that local abundance of noctuid moths in olive orchards may be correlated to life-history traits and biological characteristics of species that reflect their opportunistic condition.

## Materials and Methods

### Study site

The study was carried out in the Guadalquivir Valley (Andalusia, Spain), located from 37° 51′ N to 37° 58′ N and from 4° 15′ W to 4° 28′ W. In particular, sampling sites were located in Bujalance, province of Córdoba (30SUG79) at an altitude ca. 350 m. a. s. 1. The climate is continental Mediterranean (Capel Molina 1981): mean annual rainfall is 500 mm with hot, dry summers (29° C on average), and relatively cold and wet winters (9.5° C on average). Olive orchards comprise the main landscape (81% of the cultivated area), followed by crops such as wheat and sunflower (19%), plus some areas cultivated with fruit trees (0.02%) (Redondo et al. 1994). Olive trees are grown under an intensive regime in which emergent weeds are controlled by two additions per year of the herbicide Simazine (50%, 4 L/ha), one in March-April, another in October. Synthethic organic insecticides are used to control pests, mainly *Prays oleae* Bernard (Lepidoptera, Yponomeutidae) and *Bactrocera oleae* Gmelin (Diptera, Tephritidae): one addition of Dimethoate (40%, 150 ml/ha) for *P. oleae* in May and two treatments with the same product for the control of *B. oleae* in September and October, respectively. In addition, copper sulphate is used (40%, 1g/1) to control leaf spot diseases. Fertilizers are applied as required (urea and other foliar fertilizers in January-February). Consequently, the wild vegetation is reduced to riversides, roadsides, and the edges of some properties (for more details about community composition of this vegetation type see Redondo et al. 1994). The studied agrosystem and its managing regime are representative of the main landscape and practices in the Guadalquivir basin, so that the overall patterns arising from the data are presumed to be general (in spite of the known geographical and interannual variation in species composition and richness in Noctuidae; Luff and Woiwod 1995, Summerville and Crist 2008).

### Abundance data

In this paper, attention has been focused on Noctuidae for a number of reasons. They are numerically important, both by their great diversity and abundance (Holloway 1992; Yela 1998; Ramos et al. 2001; Novotny et al. 2006), so that they usually comprise a major proportion of captures at light traps (see e.g. Janzen 1988; Barlow and Woiwod 1989; Holloway 1992). They are also ubiquitous, living in all kinds of terrestrial biotopes, and are good indicators of biodiversity in given areas (Morrone and Ruggiero 2001; Summerville et al. 2004; Scalercio et al. 2008). They are also important food sources for other organisms as bats, birds, and insect parasitoids, establishing complex interactions with them (Jacobs et al. 2008; Jones et al. 2008; Gassmann et al. 2010, and references therein). In general, they manifest rapid response to environmental perturbations (Erhardt and Thomas 1991; Luff and Woiwod 1995), which reflects well on their functional significance (Holloway 1992). A number of species produce major agricultural and silvicultural impact because their larvae are pests of huge significance (Bourgogne 1951; Cayrol 1972; Gómez Bustillo et al. 1986; Holloway et al. 1992; Baragaño et al. 1998). Additionlly, census methods are simple and inexpensive (Yela 1992; Scalercio et al. 2008).

For collecting moths, light traps were used which are considered one of the best methods to register adults of a wide range of noctuid species (Williams 1936; Löbel 1982; Muirhead-Thomson 1991). In particular, hand- and net-sampling was done using five 250 W mercury vapor bulbs (Phillips H37KC-*250/DX,* www.philips.com) put in front of white sheets, as described in the literature (e.g. Yela 1992), situated 30 m apart from each other and placed in same sites all the time. In the conditions of our study, attraction radius should reach not more than 30 m (e.g. Yela 1992; Yela and Holyoak 1997; and references therein). One (and the same) observer collected all adult noctuids that arrived to the sheets during the first three hours every night, that is by far the period of maximal activity (Yela and Holyoak 1997). Because numbers of collected moths were usually low on each bulb, total captures were pooled together. As a whole, adults of 66 species were detected over three years of weekly samplings (1987–1989). Originally, numbers of individuals followed a polynomial distribution, indicating a very unstable structure of the noctuid assemblage (which is expected for a highly modified and managed ecosystem). Therefore, for data analysis, numbers of individuals per species and per year were log-transformed to meet the assumption of normality (Zar 1984). There could be differences among species in the number of captured individuals caused by differential attraction of the light trap (Muirhead-Thomson 1991; Yela and Holyoak 1997); but usually, it is assumed that this fact does not have a significant effect on the abundance patterns (Taylor and Carter 1961; Taylor and Woiwod 1980; Taylor 1986; Quinn et al. 1997). Additionally, in order to explore potential effects of environmental factors on sampling (Williams 1940, 1961; Hardwick 1972; Pearson 1976; Gaydecki 1984; Dent and Pawar 1988; Yela and Holyoak 1997), data for temperature, moonlight, cloud cover, and wind were recorded. Only temperature and moonlight light showed some effect (r^2^= 0.32; P < 0.001 and r^2^ = 0.034; P < 0.01), being moderately positive and slightly negative, respectively (Pérez-Guerrero et al. *in prep*). Appendix 1 shows the whole census by species.

### Biological characteristics

For each species found, six relevant biological characteristics were selected and were categorized as in Quinn et al. (1997). Characteristics include relevant life-history traits (number of eggs, number of generations per year, dispersal ability, feeding specificity) and other important ecological features (plant type of larval host plant and distribution type). Most of these traits are subject to geographic variability; however, categorical levels of variables have enough range to cope with this variability. Categories of the 66 species evaluated are also shown in the Appendix 1. Body size was not considered as a covariable since the adults of most of the species studied showed relatively similar size, so that intraspecific variation did not significantly differ from interspecific variation (F_6_0,4220 = 0.93; P = 0.65);

### Life-history traits

**Number of eggs.** Data are derived from a dataset compiled during more than 30 years (Yela, unpublished data). They were obtained mainly by dissecting female abdomina after boiling them with KOH (during the process of genitalia preparation) and counting all the forming eggs in the ovarioles under a standard binocular microscope. Number of examined females varies greatly from species to species; therefore, our data was pooled with that obtained from the bibliography (which must be considered very cautiously). This produces a rough estimation, based on which three categories were considered: from 1 to 100 (1), from 101 to 500 (2), and more than 500 eggs (3).

**Number of generations per year.** Species that complete one generation per life cycle (1; univoltine species), two generations (2; bivoline species), or more than two generations (3; multivoltine species) were classified according to Bergmann (1954), Meszaros (1967), Beck (1960), Ortiz and Templado (1982), Bembenek and Krause (1984), and Yela (1992).

**Dispersal ability.** Based mainly on Yela (1992) and on other authors such as Koch (1964), French (1969), Malicky (1967 and 1969), Mikkola (1970), and Eitschenberger and Steiniger (1973) we distinguished low-mobility species, of which adults move around 150–500 m and fly relatively low (1); high-mobility species, of which adults fly higher and may reach as far as 1 km daily, sometimes displaying strong intraareal displacements (2); and migratory species, which travel long distances recurrently (3).

**Feeding specificity.** Species were divided into three feeding-specificity categories according to an increasing degree of polyphagy: monophagous, species feeding on one plant genus only (1); oligophagous, feeding on one plant family (2); and broad polyphagous, feeding on several plants families (3). Classification criteria were based on Allan (1949), Bergmann (1954), Beck (1960), Seppänen (1970), Forster and Wohlfahrt (1960, 1971), Balachowsky (1972), Meszaros (1972, 1974), Carter (1979), Patocka (1980), Hacker (1989), Sauer (1982), Heath and Emmet (1979, 1983), Koch (1984), Merzheevskaya (1989), Fibiger (1990, 1993), Yela (1992), Ronkay and Ronkay (1994, 1995), Ronkay et al. (2001), Hacker et al. (2002), Goater et al. (2003), Zilli et al. (2005), Fibiger and Hacker (2007), and Ahola and Silvonen (2008).

**Plant type of larval host plant.** Plant type was either herbaceous (1) or woody (2) (Allan 1949; Bergmann 1954; Beck 1960; Seppänen 1970; Forster and Wohlfahrt 1960, 1971; Balachowsky 1972; Meszaros 1972, 1974; Carter 1979; Patocka 1980; Heath and Emmet 1979, 1983; Sauer 1982; Koch 1984; Hacker 1989; Merzheevskaya 1989; Yela 1992; Ahola and Silvonen 2008; the authors' own data was also used).

### Species range or distribution type

Taking into account arguments and data in Boursin (1964, 1965), Calle (1974, 1983), Fibiger (1990, 1993), Yela (1992), Ronkay and Ronkay (1994, 1995), Ronkay et al. (2001), Hacker et al. (2002), Goater et al. (2003), Zilli et al. (2005), and Fibiger and Hacker (2007) species were classified as Northern (1), Mediterranean (2), or Tropical-Subtropical (3) according to the baricenter of the species' range.

### Habitat

Based on larval trophic preferences, the habitat of each species is indicated. Distinguishing species were associated with agro-ecosystems (A), grasslands (G), shrublands (S), and woodland (W) (Rejmánek and Spitzer 1982).

### Statistical analysis

**Species ordination.** Categorical Principal Components Analysis was used to evaluate whether biological traits selected were related to habitats with which species are associated. “Princal” module of SPSS v 13 program (SPSS Inc. www.spss.com) was used for the analysis.

**Comparative analysis.** Phylogenetic effects may influence relationships between local abundance and life-history traits. Phylogenetically related species may share several traits; consequently, if one trait is correlated to abundance, other traits shared by this species are also correlated to abundance. This is actually the case in our dataset, so that an examination without taking phylogeny into account resulted in significant effects of every factor considered (Pérez-Guerrero 2001). Thus, a phylogenetically controlled comparative method is needed (Harvey 1996). One of the most frequently used methods to control for phylogeny in comparative studies is phylogenetic independent contrasts (PICs) (Harvey and Pagel 1991). PICs compare attributes of species differing in a specific phenotype within a given taxon level. Each PIC is a different fork in the evolutionary tree, so the comparison within a PIC is independent of the comparison in another PIC. In this paper, relationships between local abundance, life-history traits, and distribution type of an olive-orchard noctuid moth assemblage were analyzed while controlling for phylogenetic effects. The program, CAIC (Comparative Analysis by Independent Contrast, Purvis and Rambaut 1995), was used for the analysis. CAIC requires knowing the phylogeny and branch length. In the absence of a generally accepted detailed phylogeny for Noctuidae (see discussions in Yela 1998; Yela and Kitching 1999; Mitchell et al. 2000; Lafontaine and Fibiger 2006), taxonomic classification of this family was used (based in Lafontaine and Fibiger 2006) assuming that taxonomy reflects phylogeny (Purvis and Rambaut 1995). A direct consequence of this assumption is that all branches in the phylogeny tree are of equal length, i.e. a punctual model of evolution which may produce type I errors (that are assumed independently of the phylogenetic determination; see Purvis et al. 1994, but see also Martins 1996 or Abouheif 1999 for critiques).

In order to examine whether there were differences in abundance among taxa, BRUNCH option included in CAIC was used. BRUNCH takes categorical variables as the predictors and abundance as a continuous variable. If there is no trend between different taxa with respect to the different categories, the average of the contrasts made with BRUNCH for abundance will not differ significantly from zero. The trend was evaluated with one sample t test (Purvis and Rambaut 1995). The sign of the average value reflects the trend of the abundance vs. biological characteristics relationship. To control for a potential effect of migrant, allochthonous moths two analyses were performed, either excluding migratory species or including all species detected (Quinn et al. 1997).

## Results

### Species ordering

**Figure 1.**
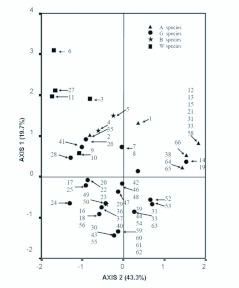
Order of 66 species according to life-history traits. Names and numbers of species are given in the Appendix 1 (A: agrosystem species; G: grassland species; S: shrubland species; W: woodland species). High quality figures are available online.

The ordering of the 66 species with respect to biological characteristics is shown in Figure 1. The first two PCA axes explained over 63% of the variation observed in the data. The most correlated life-history traits with the first two PCA axes were number of eggs (ρ= 0.85 and 0.35, respectively) and dispersal ability (ρ= 0.76 and -0.364, respectively). The analysis differentiated species association with regard to defined habitats, indicating that biological features were related to these associations. All species associated with the agro-ecosystem except *Sesamia nonagroides* Lef. were grouped next to *Agrotis spinifera* L. A second group containing more species, which were associated with grassland, showed a scattered distribution in the graph and formed small subgroups. The third group was located in the centre of the plot as it was more heterogeneous and comprised of species associated with shrubs, half of the scarce woodland species, and some grassland species together with *S. nonagrioides.* Finally, the other half of the woodland species appeared far away from the rest in the plot (Figure 1).

### Comparative analysis

There were no significant differences in abundance among habitat-associated groups (Table 1). However, abundance varied significantly among species with different dispersal ability and number of generations per year. The positive relationship of these life-history traits with abundance showed that highly mobile, multivoltine taxa were more abundant locally (Table 1).

Analysis of the sample without migratory species (53 remaining species) showed that dispersal ability and number of generations were again related to abundance patterns (Table 2). Once again, the positive relationships showed higher abundance for the species with several generations per year and greater dispersal capacity. It is important to emphasize that only two multivoltine species remained in this analysis. When these species were removed from the analysis (leaving 24 univoltine and 27 bivoltine species), the result was non-significant (t = 2.05; df = 15; P > 0.05).

**Table 1.**
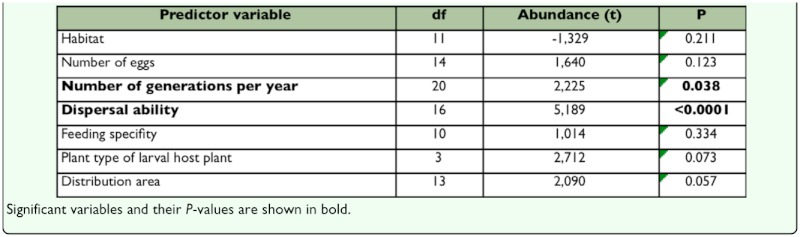
Results of one sample T-test comparing local abundance of 66 species included in the sample for the six life-history traits and habitat (defined on Materials and Methods section).

**Table 2.**
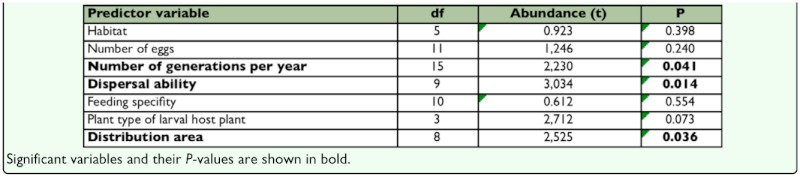
Results of one sample T-test comparing local abundace of 53 non-migrant species included in the sample for the six life history traits and habitat (defined on Materials and Methods section).

Moreover, the results showed that distribution type also determined differences in abundance for the remaining 53 species (Table 2). It is worth noting that the two multivoltine species were the only species with a tropical-subtropical distribution, the northernmost stable populations of which reach the south of Spain. The analysis showed significant differences in abundance when these species were removed (t = 2.5; df = 7; P < 0.05), revealing that Mediterranean taxa were more abundant than northern ones. The rest of variables showed no significant variation with abundance.

## Discussion

The ordering of the species according to life history traits, habitat, and chorological pattern showed that, at the local scale, there is an association of some of these variables with species abundance so that the axes of the PCA explained 63% of the variation of abundance. Nevertheless, surprisingly no differences in abundance between species associated with different habitats were found, in contrast to Brown (1984), Inkinen (1994), and Quinn et al. (1997), showing that, altogether, life history traits alone do not explain differences in local abundance in olive orchards (despite the clear majority of grassland species). Only singular traits explained the differences. To some extent, this may be an artifact due to the characterisation of the variable ‘habitat’ which does not inform on the range of habitats used by each species, but rather on the main type of habitat used. A few individuals of a few woodland species most likely owe their presence to the remaining riparian forest associated with streams beneath the olive orchards. These results support the idea that conservation of riparian forest has capital consequences for the maintenance of particular species and thus for biodiversity in olive orchard landscapes. Thus, vegetation growing along and beneath rivers and creeks would be worth preserving, in order to maximize the probability of survival of local populations of noctuids associated with hardwood vegetation and, more generally, to maintain higher levels of biodiversity. This may be relevant bearing in mind the functional role of noctuids as prey and hosts (Holloway 1992; Jacobs et al. 2008; Jones et al. 2008; Gassmann et al. 2010 and references therein). Although this research focused on a local pattern, patterns of larger spatial scale in agro-ecosystem can be a further challenge since determinants of abundance may vary depending on scale (Gaston 1994; Brown et al. 1995, 1996; Freckleton et al. 2006; Zuckerberg et al. 2009).

The general patterns found by Inkinen (1994) and Quinn et al. (1997) revealed that variations of trophic traits are associated with differences in abundance. Gaston and Lawton (1988a) found similar results for bracken herbivores. Nevertheless, for a singular agroecosystem such as olive orchards, no relationship was found between trophic traits and abundance (Tables 1 and 2). The results show that the most abundant taxa in olive orchards have a higher dispersal ablility and are able to complete several generations throughout the year. Most noctuid species (except those associated with trees and shurbs) have herbaceous plants (neighbouring plants or “weeds”) as the principal food resource. In olive orchards, due to the type of management, this resource changes dramatically over time (seasonal, ephemeral plants) and space since the herbicide does not cover the whole crop, allowing patches of herbaceous plants to remain (edges of ways, ditches, etc.). Therefore, noctuid species with higher dispersal ability or with a versatile life cycle (facultative multivoltine species) would have more chances to access to food plants and thus have higher resource availability as opposed to the other species. Thus, according to our results and hypotheses of other authors (Blackburn et al. 1996; Blackburn et al. 1997; Gregory and Gaston 2000), species with higher dispersal ability and several generations per year would be more abundance in olive orchards.

When the effect of migrant species was removed (Table 2), the species distribution type also explained differences in abundance. It should be noted that most of the migrant species have a basically tropical-subtropical distribution type; therefore, extra-areal migratory fronts reaching Europe recurrently may mask the result regarding distribution. Once controlled for this effect, results showed higher abundance for Mediterranean taxa than for more northern ones. Given this differential trait and according to the core of their geograpical range (see Materials and Methods section), more abundant species would be those with the baricenter of their geographical range closer to the study site, i.e. Mediterranean species. The study populations of tropical-subtropical and northern (Euro-Asiatic) species are located closer of the edge of their respective geographical ranges. This result would be consistent with the large-scale pattern according to which, considering the entire geographical range of a species, the average local abundance tends to peak towards the core and decline towards the periphery (Hengeveld and Haeck 1981; Brown 1984; Lawton 1993). Several studies showed results following this rule (Hengeveld and Haeck 1981, 1982; Svensson 1992; Tellería and Santos 1993; Brown et al. 1995, 1996; Guo et al. 2005; Antonovics et al. 2006), although other authors (Blackburn et al. 1999; Freckleton et al. 2006; Sagarin et al. 2006; Wilson 2008) found results revealing the controversy of this pattern and the effect of sampling effort.

Therefore, we conclude that the association of the noctuid species to the different habitats is not related to differences in local abundance. Olive-orchard characteristics seem to modulate the general local abundance pattern of noctuids moths, and trophic traits do not explain abundance variation within taxa. In contrast, dispersal ability and number of generations per year explain this variation and support a higher local abundance range. Mediterranean taxa are the most abundant species, revealing a narrow relation between this kind of species, the habitat, and its requirements.

In any case, we have to stress out that our study is, to some degree, a first attempt to take on the issue and that long term monitoring would be necessary to clearly separate external causes of abundance variation (e.g. Mutshinda et al. 2007) from variation in population density depending from biological processes that may be even totally unpredictable (e.g. Beninca et al. 2008).

## Figures and Tables

**Appendix 1.**
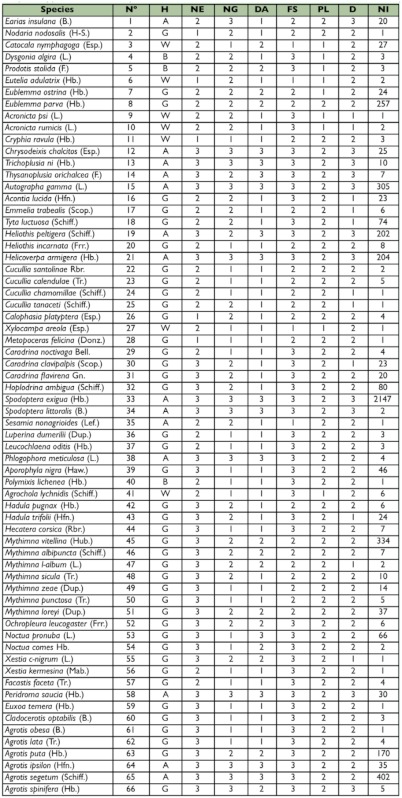
Categories of life-history traits for 66 species evaluated (see Materials and Methods). N° is the identification number of the species, H habitat of the species, NE number of eggs, NG number of generations per year, DA dispersal ability, FS Feeding specifity, PL plant type of larval host plant, D distribution and NI number of individuals observed per species.
